# Reactivation of Parvovirus B19 Infection: An Uncommon Trigger of Macrophage Activation Syndrome in Adult-Onset Still’s Disease

**DOI:** 10.7759/cureus.37231

**Published:** 2023-04-06

**Authors:** Natnicha Leelaviwat, Sabiha Armin, Poemlarp Mekraksakit, Kenneth Nugent

**Affiliations:** 1 Internal Medicine, Texas Tech University Health Sciences Center, Lubbock, USA; 2 Internal Medicine, University of Texas Health Science Center at Houston, Houston, USA; 3 Nephrology and Hypertension, Mayo Clinic, Rochester, USA; 4 Internal Medicine/Pulmonary and Critical Care Medicine, Texas Tech University Health Sciences Center, Lubbock, USA

**Keywords:** fever of unknown origin, hemophagocytic lymphocytic histiocytosis, adult-onset still’s disease, macrophage activation syndrome, parvovirus b19

## Abstract

A 40-year-old woman presented with four weeks of intermittent high-grade fever, cough, and joint pain, and two weeks of a generalized rash. She was found to have adult-onset Still’s disease (AOSD) and rapidly developed macrophage activation syndrome (MAS) on the second day of admission. Among infectious etiologies, Epstein-Barr virus and members of the herpes virus family are common triggers of MAS. However, our patient was found to have reactivation/recurrence of parvovirus B19 infection as the cause; this is an uncommon trigger reported infrequently in the medical literature. Despite intensive treatment, the patient passed away.

## Introduction

Macrophage activation syndrome (MAS) is a form of hemophagocytic lymphocytic histiocytosis (HLH), which is a rare and life-threatening systemic inflammatory disorder that can occur in rheumatologic disorders. It is most commonly seen in systemic juvenile idiopathic arthritis (sJIA) in children but is often underrecognized in Kawasaki disease, systemic lupus erythematosus, and adult-onset Still’s disease (AOSD) [1]. The mortality rate of MAS ranges between 20-42% [2]. This diagnosis is always challenging given the non-specific presentation and lack of definitive criteria, especially in adults in whom it is an uncommon syndrome. Due to the rapid progression and high mortality with MAS/HLH, clinical suspicion is the key to early diagnosis and treatment. We report a case of a patient with AOSD who had reactivation/recurrence of parvovirus B19 infection and then rapidly developed MAS with multiorgan failure. 

## Case presentation

**Figure 1:**
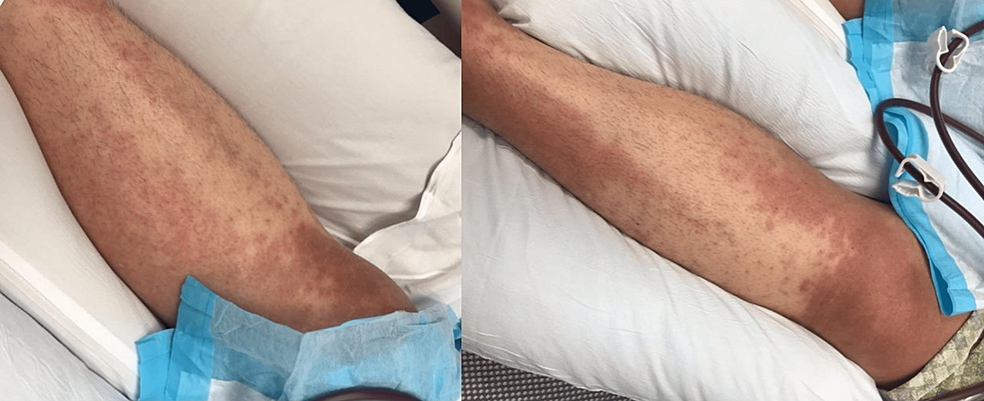
Diffuse, blanchable, salmon-color macular rash on lower extremities

A 40-year-old woman with hypertension, transient ischemic attacks, and a history of squamous cell cancer of the tonsil in remission presented to the emergency department with complaints of four weeks of intermittent high-grade fever, dry cough, polyarticular and non-migratory joint pain involving knees, wrists, ankles, and elbows, and two weeks of a generalized rash on the trunk and lower extremities (Figure 1). The rash usually came with a fever and went away when she was afebrile.

On examination, she had a fever of 103.4 °F, splenomegaly, and a diffuse, blanchable, salmon-colored macular rash. Initial laboratory tests showed neutrophilic leukocytosis (28,880/µL, polymorphonuclear leukocytes (PMN) 90%), thrombocytosis (515,000/µL), transaminitis (Aspartate transaminase (AST) 190 intl units/L; alanine transaminase (ALT) 43 intl units/L), and elevated erythrocyte sedimentation rate (ESR) 70 mm/hr and C-reactive protein (CRP) 19.6 mg/dL (Table 1).

**Table 1:** Laboratory test results

Investigation	Laboratory test results	Reference range
White blood cell (K/µL)	28.88 (neutrophils 90%, lymphocytes 0.8%, eosinophils 0.8%, band 2.5%)	3.98-10.04
Hemoglobin (g/dL)	14.2	11.2-15.7
Platelet (K/ µL)	515	182-369
Serum creatinine (mg/dL)	4.6	0.5-1.2
Sodium (MMOL/L)	141	136-145
Potassium (MMOL/L)	4.6	3.5-5.1
Aspartate transaminase (intl units/L)	190	5-37
Alanine transaminase (intl units/L)	43	5-41
Erythrocyte sedimentation rate (mm/hr)	73	0-20
C-reactive protein (mg/dL)	19.6	0-0.5
Antinuclear antibody	Negative	Negative
Rheumatoid factor	Negative	Negative
Cyclic citrulline peptide (units/mL)	<0.5	<3
Flow cytometry obtained from blood	Negative	Negative
Serum beta-human chorionic gonadotropin	Negative	Negative
Interleukin 2R (pg/mL)	33950	< 2515
Prothrombin time (sec)	231	9.4-12.5
Partial thromboplastin time (sec)	30.5	25-36.5
Fibrinogen level (mg/dL)	174	200-393
D-dimer (ng/mL)	61243	< 500
Lactate dehydrogenase (units/L)	1878	135-225
Ferritin level (ng/mL)	>100,000	13-150
Antiphospholipid panel	Negative	Negative
Triglyceride (mg/dL)	584	50-200

She was started on empiric vancomycin and cefepime. Her white blood cell count, platelet count, and liver enzymes three months prior to admission were normal. The next day, the patient’s mentation deteriorated, and she required intubation. Magnetic resonance imaging of the head and electroencephalography were unremarkable. A lumbar puncture revealed lymphocytic pleocytosis (WBC 117/mm3, lymphocytes 78%) and a high CSF protein (100 mg/dL). CSF glucose and opening pressure were normal. All the cultures came back negative, and bacterial infection seemed less likely as the cause. Empiric acyclovir and doxycycline were added for possible herpes or spirochetes infection.

Despite fluid resuscitation, the patient developed worsening acute kidney injury and was started on renal replacement therapy. Hematologic malignancy/solid organ tumor was unlikely since flow cytometry obtained from blood was negative, and whole-body CT scans did not show any masses. Drug reaction with eosinophilia and systemic symptoms (DRESS) was a possibility, but the patient ingested only acetaminophen and ibuprofen for the past four weeks. Her rash was not typical for DRESS, a CBC did not show eosinophilia, and the skin biopsy was not consistent with DRESS. Vasculitis and autoimmune disorders were unlikely since the ANA and rheumatoid factor were negative, and the skin biopsy result did not have vasculitis features. So, MAS/HLH was likely since the patient met all four Yamaguchi major criteria for diagnosis of AOSD and three minor criteria (Table 2).

**Table 2:** Yamaguchi criteria: the Yamaguchi criteria require the presence of five features, with at least two being major diagnostic criteria after excluding infection, malignancy, or other rheumatic disorders known to mimic AOSD AOSD: adult-onset Still’s disease

Yamaguchi criteria for AOSD
The four major Yamaguchi criteria
Fever of at least 39ºC (102.2ºF) lasting at least one week
Arthralgias or arthritis lasting two weeks or longer
A nonpruritic macular or maculopapular skin rash that is salmon-colored in appearance and usually found over the trunk or extremities during febrile episodes
Leukocytosis (10,000/µL or greater), with at least 80 percent granulocytes
The five minor Yamaguchi criteria
Sore throat
Lymphadenopathy
Hepatomegaly or splenomegaly
Abnormal liver function studies, particularly elevations in aspartate and alanine aminotransferase and lactate dehydrogenase concentrations
Negative tests for antinuclear antibody (ANA) and rheumatoid factor (RF)

Additional laboratory tests (Table 1) revealed elevated triglycerides (584 mg/dL), ferritin (>100,000 ng/mL), soluble interleukin-2 receptor (33950 pg/mL), and low fibrinogen (174 mg/dL); she met six out of eight criteria for HLH which included fever ≥38.5°C, splenomegaly, hypertriglyceridemia, hypofibrinogenemia, and elevated ferritin and soluble interleukin-2 receptor (Table 3).

**Table 3:** HLH-2004 diagnostic criteria HLH: hemophagocytic lymphohistiocytosis; NK cell: natural killer cell; IL: interleukin

HLH-2004 diagnostic criteria
A molecular diagnostic consistent with HLH
Diagnostic criteria for HLH fulfilled (five out of the eight criteria below)
Fever
Splenomegaly
Cytopenia, with at least two of the following:
Hemoglobin <9 g/dL (for infants <4 weeks, hemoglobin <10 g/dL
Platelets <100,000/ µL
Absolute neutrophil count <1000/ µL
Hypertriglyceridemia (fasting triglycerides >265 mg/dL) and/or hypofibrinogenemia (fibrinogen <150 mg/dL)
Hemophagocytosis in bone marrow, spleen, lymph node, or liver
Low or absent NK cell activity
Ferritin >500 ng/mL
Elevated soluble CD25 (soluble IL-2 receptor alpha [sIL-2R]) two standard deviations above age-adjusted laboratory-specific norms

Bone marrow biopsy results revealed morphologic features of hemophagocytosis. Moreover, the calculated HScore for the patient was 270 points indicating > 99% probability of HLH (Table 4) [3].

**Table 4:** HScore for reactive hemophagocytic syndrome

Parameters	No. of points (criteria for scoring)
Known underlying immunosuppression	0 (no)
	18 (yes)
Temperature (°C)	0 (<38.4)
	33 (38.4–39.4)
	49 (>39.4)
Organomegaly	0 (no)
	23 (hepatomegaly or splenomegaly)
	38 (hepatomegaly and splenomegaly)
No. of cytopenias	0 (1 lineage)
	24 (2 lineages)
	34 (3 lineages)
Ferritin (ng/ml)	0 (<2,000)
	35 (2,000–6,000)
	50 (>6,000)
Triglyceride (mmoles/liter)	0 (<1.5)
	44 (1.5–4)
	64 (>4)
Fibrinogen (gm/liter)	0 (>2.5)
	30 (≤2.5)
Serum glutamic oxaloacetic transaminase (IU/liter)	0 (<30)
	19 (≥30)
Hemophagocytosis features on bone marrow aspirate	0 (no)
	35 (yes)

Work-up for infections on the third day of admission revealed a positive serum parvovirus-B19 PCR and IgG with a negative IgM (Table 5).

**Table 5:** Parvovirus serology

Parvovirus serology	Day 3 of admission	Day 13 of admission
Parvovirus B-19 IgM	Negative	Negative
Parvovirus B-19 IgG	Positive	Positive
Parvovirus B-19 DNA PCR	Detected	Not detected

Serology tests for CMV were negative; EBV IgG was positive and EBV IgM was negative (Table 6).

**Table 6:** Infectious workup and serology Ig: immunoglobulin; VZV: varicella-zoster virus; HSV: herpes simplex virus; PCR: polymerase chain reaction; CSF: cerebrospinal fluid

Infectious workup	Results
HIV	Non-reactive
Viral hepatitis profile	
Hepatitis A IgG	Positive
Hepatitis A IgM	Negative
Hepatitis B core IgG	Negative
Hepatitis B Surface Ab	Negative
Hepatitis B Surface Ag	Negative
Hepatitis C IgG	Negative
SARS-CoV-2 IgG	Negative
Syphilis screen	Non-reactive
CMV IgG	Negative
CMV IgM	Negative
EBV IgG viral capsid antigen antibodies	Positive
EBV IgM viral capsid antigen antibodies, IgG nuclear antigen antibodies	Negative
Mononuclear screen	Negative
VZV	IgG positive
Measles	IgG positive
HSV 1 and 2 PCR	Not detected
Rubeola	IgM < 1:20
Urine histoplasma galactomannan antigen	< 0.2
Blood culture	No growth
CSF culture	No growth
Urine culture	No growth
Cryptococcal antigen	Negative

On repeat serology tests 10 days later, the parvovirus PCR was negative, but the parvovirus IgG remained positive consistent with reactivation/recurrence of parvovirus-B19 viremia, which is an uncommon event.

The patient was started on high-dose intravenous methylprednisone on the second day of admission due to high clinical suspicion of HLH/MAS even though multiple test results had not come back. Etoposide per HLH94 protocol was started on the fifth day of admission after the diagnosis of HLH/MAS was confirmed. After the second dose of etoposide, the patient was more responsive. However, she developed severe pancytopenia, most likely a side effect of etoposide, and her hemodynamic status did not improve. Her family requested palliative care only, and she died one day after transitioning to comfort care.

## Discussion

Adult-onset Still’s disease is a rare systemic inflammatory disorder of unknown etiology characterized by spiking high fevers, arthritis or arthralgia, maculopapular salmon-colored rash, neutrophilic leukocytosis, and hyperferritinemia [4]. The Yamaguchi criteria is the most commonly used criteria for the diagnosis and have a sensitivity of 96.2% and specificity of 92.1% [5]. Macrophage activation syndrome/ hemophagocytic lymphocytic histiocytosis is the most serious and life-threatening complication of AOSD and is a dysregulated immune state possibly caused by abnormal downregulation of activated macrophages and lymphocytes leading to excessive cytokine production. It can occur as a primary/familial disorder or secondary disorder that is triggered by infection, malignancy, an autoimmune disease, or drugs. The diagnosis is based on the published diagnostic criteria used in the HLH-2004 trial which required at least five out of eight criteria (Table 3) [6]. However, the HLH-2004 criteria were first developed for pediatric patients and may have some limitations in adult patients. Therefore, other classification/diagnostic tools have been developed. The HScore was developed to identify acquired HLH in adults, and a score of ≥ 250 indicated > 99% probabilities of HLH (a cut-off of 164 has a sensitivity of 100% and specificity of 89.9%) (Table 4) [3]. The MS score (Table 7) was developed to discriminate between MAS and active sJIA [7] and is under investigation for application in AOSD patients [8,9]. The calculated MS score for our patient was 10.807, and a score ≥ −2.1 indicates MAS with a sensitivity of 100% and specificity of 29.85% [10]. However, this score has not been validated in patients with AOSD. The frequency of AOSD patients who develop MAS can be as high as 10-15%, and patients may present with MAS at the time of diagnosis or during the disease course [11].

**Table 7:** Best-fitted model with the best combination of clinical and laboratory variables and β-coefficients used for the calculation of the MS score

The MS score parameter	β-coefficient	95% CI
Central nervous system (CNS) involvement	2.44	1.26 to 3.65
Hemorrhagic manifestations	1.54	0.00 to 3.05
Active arthritis	−1.30	−2.05 to 0.00
Platelet count (PLT), x 109 /L	−0.003	−0.005 to −0.001
Lactic dehydrogenase (LDH), U/L	0.001	0.0002 to 0.002
Fibrinogen, mg/dL	−0.004	−0.006 to −0.002
Ferritin, ng/mL	0.0001	0.00 to 0.0002

The etiology of MAS is unclear. However, infection, malignancy, genetic predisposition, medications, and flares of AOSD have been reported as potential triggers of MAS in AOSD [12]. The herpes virus family, including EBV, CMV, HSV, and varicella zoster, are the most common viral triggers [11]. Parvovirus-B19 has been reported as a less common trigger. However, our patient’s serology results were consistent with the reactivation/recurrence of parvovirus-B19 infection as her trigger for MAS, which has been infrequently reported in the medical literature. 

Parvoviruses are small, nonenveloped viruses with single-stranded DNA that has approximately 5000 nucleotides [13]. Transmission is predominantly by respiratory secretions but can occur through blood transfusion [14]. Adults typically present with arthralgias and possibly a macular rash. Some patients develop transient aplastic crises. This virus can also cause hemophagocytic syndromes, hepatitis, vasculitis, myocarditis, glomerulosclerosis, and meningitis. The diagnosis depends on the detection of parvovirus-B19 IgM antibodies and a PCR test for the DNA. Viremia occurs one week after exposure and lasts about five days, whereas IgM antibodies can be detected 10 to 14 days after infection and can persist for up to five months; IgG antibodies appear about 15 days after infection and remain high for several months. Chronic infection can occur, especially in immunocompromised patients. Positive IgG serology does not necessarily mean protection [15]. Hemophagocytic lymphocytic histiocytosis triggered by reactivation and recurrence of parvovirus B-19 was previously reported. Orth et al. reported recurrent parvovirus B-19 viremia resulting in two episodes of HLH in a 25-year-old patient who presented with a three-week history of high fever. HLH was diagnosed based on HLH-2004 criteria and HScore, and the patient responded to the treatment with corticosteroids, etoposide, and intravenous immune globulin [16]. Karrasch et al. reported probable parvovirus B-19 reactivation and primary EBV infection resulting in HLH and fulminant hepatitis in a 27-year-old patient who was admitted to the hospital after having fever, fatigue, and jaundice for two days. HLH was diagnosed based on HLH-2004 criteria. This patient had a good clinical outcome after high-dose corticosteroid treatment likely due to an early onset form of HLH [17].

Unfortunately, due to the lack of controlled studies on the treatment, management is largely empiric. The treatment of MAS is different from that recommended for HLH. The mainstay of therapy is immediate high-dose corticosteroids with methylprednisolone (1 g daily) for three to five consecutive days [11]. Cyclosporin (2-7 mg/kg daily) can be added in patients with an inadequate immediate response, as well as an IL-1-blocking agent. In patients with severe active disease or CNS involvement, a reduced dose of etoposide (50-100 mg/m2 once weekly) can be very effective [18]. Studies with anakinra, cyclosporin, etoposide, plasma exchange, and immunoglobulin are conflicting. Investigation of malignancy should prompt malignancy-specific treatment [11]. In EBV-associated HLH, rituximab can be used as an additional treatment, and in HIV and malignancy-associated HLH, etoposide can sometimes be helpful. There are no studies on specific treatments for parvovirus B19-associated MAS.

## Conclusions

Patients with HLH/MAS can rapidly decline. Early clinical suspicion is the key to prompt investigation and treatment. Thus, HLH/MAS should be one of the differential diagnoses in patients who present with sepsis-like symptoms. High-dose intravenous corticosteroids should be started by the time of diagnosis without delay. Reactivation/recurrence of parvovirus-B19 infection can be one of the uncommon triggers of developing HLH/MAS.
